# A comparison study of insulin concentrations in follicular fluid, serum and in vitro-production of bovine embryos – risks of generating unfavourable metabolic conditions during early development

**DOI:** 10.1186/1751-0147-57-S1-P5

**Published:** 2015-09-25

**Authors:** Denise Laskowski, Ylva Sjunnesson, Hans Gustafsson, Patrice Humblot, Göran Andersson, Renée Båge

**Affiliations:** 1 Department of Clinical Sciences, Swedish University of Agricultural Sciences, Uppsala, Sweden; 2 Department of Animal Breeding and Genetics, Swedish University of Agricultural Sciences, Uppsala, Sweden

## Introduction

Insulin has frequently been used as a stimulatory factor in routine in vitro embryo production (IVP) and is added in supra-physiological concentrations to different media. Meanwhile, insulin as a key metabolic hormone is elevated in patients with metabolic syndrome or diabetes, syndromes known to impair fertility.

## Objective

This study compared in vitro-used and in vivo-measured insulin concentrations and elucidates explanations and risks for this difference.

## Methods and results

Eleven in vivo-studies measuring insulin in serum or follicular fluid have been compared to nine IVP-protocols using insulin. In vitro concentrations were much higher than in vivo while not much is known about the different activity and stability of insulin in vitro versus in vivo. We measured insulin concentrations before and after maturation in an experimental trial using a quantitative ELISA (Mercodia bovine insulin ELISA Immunoassay) and can report stable quantities after 22 hours of incubation.

## Conclusion

The uncritical use of supra-physiological insulin levels in vitro might have negative consequences for the developing embryo as insulin has direct effects on metabolism and could even influence the epigenetic programming of the metabolism with unknown consequences for the offspring later in life. Still, insulin may react differently in in vitro models where one explanation is different stability or activity of insulin. More precise dose effect studies have to be done to draw conclusions about consequences of the use of as high doses.

